# Supplementary figures and images for: Optimization of Cyclohexanol and Cyclohexanone Yield in the Photocatalytic Oxofunctionalization of Cyclohexane over Degussa P-25 under Visible Light
Source: Molecules. 2019 Jun 15;24(12):2244. doi: 10.3390/molecules24122244 (PMC6630937; doi:10.3390/molecules24122244)

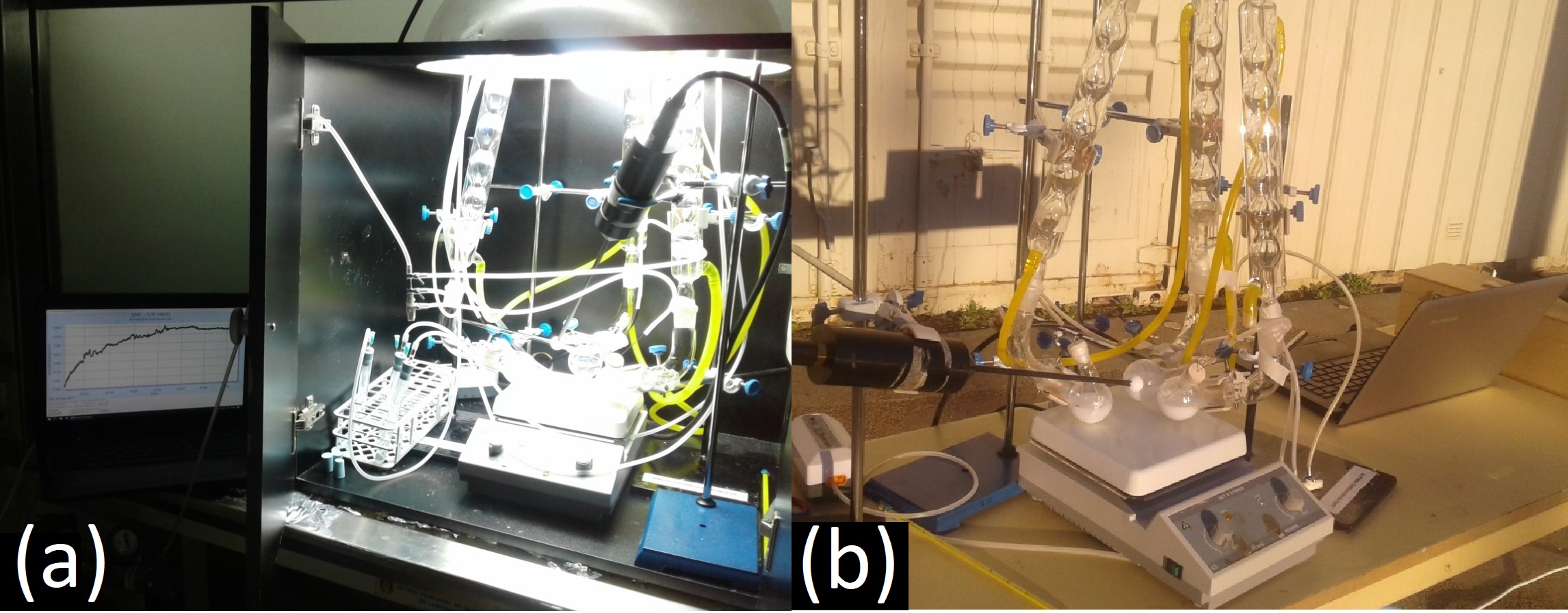

Supplement: Supplementary file 1 [file molecules-24-02244-s001.zip › molecules-514842-SI.jpg]
